# Low Ethanol Concentrations Promote Endothelial Progenitor Cell Capacity and Reparative Function

**DOI:** 10.1155/2020/4018478

**Published:** 2020-09-22

**Authors:** Lars Brodowski, Bianca Schröder-Heurich, Berina Kipke, Cara Schmidt, Constantin S. von Kaisenberg, Frauke von Versen-Höynck

**Affiliations:** ^1^Gynecology Research Unit, Hannover Medical School, Carl-Neuberg-Strasse 1, D-30625 Hannover, Germany; ^2^Department of Obstetrics and Gynecology, Hannover Medical School, Carl-Neuberg-Strasse 1, D-30625 Hannover, Germany

## Abstract

**Background:**

Endothelial progenitor cells (EPCs) are recruited to injured endothelium and contribute to its regeneration. There is evidence that moderate ethanol consumption prevents the development and progression of atherosclerosis in a variety of *in vitro* and *in vivo* models and increases the mobilization of progenitor cells. Furthermore, there are studies that identified ethanol at low concentration as a therapeutic tool to mobilize progenitor cells in peripheral blood. At the same time, the cell number of EPCs represents a close link to cardiovascular system constitution and function and contributes to cardiovascular risk. The aim of this study was to evaluate the effect of low dose ethanol on typical features of endothelial colony-forming cells (ECFCs), a proliferative subtype of EPCs.

**Methods and Results:**

We tested whether ethanol impacts the functional abilities of ECFC (e.g., migration, tube formation, and proliferation) using *in vitro* assays, the intercommunication of ECFC by exploring cell surface molecules by flow cytometry, and the expression of (anti-)angiogenic molecules by ELISA. Low concentrations of ethanol concentration promoted migration, proliferation, and tubule formation of ECFC. The expression of the cell surface marker VE-cadherin, a protein which plays an important role in cell-cell interaction, was enhanced by ethanol, while (anti-)angiogenic molecule expression was not impacted.

**Conclusion:**

Ethanol at moderate concentrations increases the angiogenic abilities of endothelial progenitor cells thus possibly contributing to vasoprotection.

## 1. Introduction

Cardiovascular disease (CVD) is a major cause of death worldwide. Most CVDs are based on atherosclerosis, a degenerative process of the arterial vascular endothelium induced by oxidative stress and chronic inflammatory status. The classic risk factors include smoking, diabetes mellitus, arterial hypertension, changes in total cholesterol, obesity, and excessive consumption of ethanol. Several studies have shown that strong ethanol consumption in adults correlates with the occurrence of CVD [1–4]. On the contrary, moderate ethanol consumption is associated with decreased morbidity and mortality from ischemic heart disease [5–7]. The protective effect of ethanol on the cardiovascular system has been attributed to the modulation of blood lipoproteins and to reduced platelet activation and thus diminished formation of thrombi. Furthermore, other studies suggest that ethanol has a direct protective effect on the myocardium [5, 8, 9].

Tissue regeneration is the focus of therapy in the treatment of CVDs. Proper vascular formation is essential in this context. Endothelial progenitor cells (EPCs) and especially their proliferative subtype endothelial colony-forming cells (ECFCs) are of pivotal importance for endothelial homeostasis and vascular remodeling. In addition, they represent a promising cell source for the revascularization of damaged tissue. The concept that alterations in EPC biology impact endothelial cell function is supported by recent studies showing that decreased numbers of circulating EPCs correlate with impaired endothelial function [10, 11]. Both a reduced number and impaired function of circulating EPCs have been observed in patients with CVD [12]. The number of circulating EPCs predicts the occurrence of cardiovascular events and can help identify patients at increased risk [13]. ECFC can be isolated from cord blood or peripheral adult blood and migrate to sites of vessel formation, possessing the ability to differentiate into mature endothelial cells, to participate in vessel repair, and to form de novo endothelium [14].

Angiogenic proteins and vascular cell adhesion proteins are important determinants of vascular health. Vascular endothelial growth factor (VEGF) has been demonstrated to promote atherosclerotic plaque progression [15]. Moreover, VEGF and placental growth factor (PIGF) stimulate endothelial cell proliferation and migration and mediate vascular growth and angiogenesis. VEGF and its soluble receptor soluble fms-like tyrosine kinase-1 (sFlt-1) are implicated in vascular damage and repair in CVD [16]. Vascular cell adhesion protein 1 (VCAM), platelet and endothelial cell adhesion molecule 1 (PECAM1), and vascular endothelial cadherin (VE-cadherin) play an important role in leukocyte extravasation, inflammatory processes, and vascular permeability and are mainly involved in the homing of cells to sites of endothelial repair and angiogenesis [17].

While in cohort studies moderate ethanol exposure is associated with reduced cardiovascular morbidity [18], little is known about the underlying pathomechanisms, specifically with regard to EPC biology. Therefore, in *in vitro* models, we investigated whether ethanol contributes to a poor EPC response or rather has a protective cardiovascular effect.

## 2. Materials and Methods

### 2.1. ECFC Isolation and Culture

ECFCs from cord blood were isolated as previously described [19]. Briefly, umbilical cord venous blood was collected immediately after delivery into sterile EDTA-coated tubes. Blood samples were centrifuged within 3 h of collection at 2,000 g for 5 min. Mononuclear cells (MCs) were isolated by density gradient centrifugation. The plasma was removed for collection and replaced with the same volume of plasma replacement buffer consisting of phosphate-buffered saline solution (PBS) supplemented with 0.025 M EDTA (Sigma-Aldrich, Steinheim, Germany or St. Louis, MO) and 1% (*v*/*v*) penicillin/streptomycin (Sigma-Aldrich). The sample volume was doubled by adding isolation buffer (PBS, 2% (*v*/*v*), fetal bovine serum (FBS, Biochrom KG, Berlin, Germany or Life Technologies, Carlsbad, CA), and 1% penicillin/streptomycin), and the sample was gently mixed. Samples were layered on Ficoll Plus (GE Healthcare, Buckinghamshire, England or Piscataway, NJ) and spun at 400 g for 40 min in a swinging bucket centrifuge with a brake in the off position. The MC fraction was collected and washed two times with an isolation buffer. Mononuclear cells were cultured in endothelial growth medium 2 (EGM-2, Lonza, Basel, Switzerland or Walkersville, MD), supplemented with supplier-recommended concentrations of human recombinant epidermal growth factor, VEGF, ascorbic acid, hydrocortisone, heparin, and recombinant insulin-like growth factor, 10% FBS and 1% penicillin/streptomycin. The MCs were plated at a density of 5 × 10^7^ cells/well on collagen-coated 6-well plates (BD Biosciences, Heidelberg, Germany, or Billerica, MA) and incubated at 37°C, 5% CO_2_. The medium was changed daily for 10 days and then every second day. The first appearance of ECFC colonies was noted as well-circumscribed monolayers of >50 rapidly proliferating, cobblestone-appearing cells. Colonies were identified by visual inspection using an inverted microscope (Olympus, Tokyo, Japan; Zeiss, Thornwood, NY). Well-defined colonies were released from the plates using cloning rings and trypsin-EDTA and collected. The cells from each separate colony were placed into a well of a collagen-coated 6-well plate and after becoming 80–90% confluent, subsequently passaged into collagen-coated T25 culture flasks. After reaching 80–90% confluence, the cells in the T25 flasks were passaged into gelatin-coated T75 flasks. At 80–90% confluence, these cells were harvested, phenotyped, and frozen in a freezing medium containing 92% FCS and 8% DMSO (Sigma-Aldrich, Steinheim, Germany).

Flow cytometric analyses to confirm the ECFC phenotype were performed using surface markers CD31, CD34, CD133, VEGFR-2, and CD45 as well as appropriate isotype controls.

All experiments were run with ECFC in passage 3 to 5 at 80–90% confluence. The concentrations of ethanol used in this study (0.5% (17 mM) and 1% (34 mM)) are doses that do not cause intoxication *in vivo* and correspond to 2 to 4 standard drinks.

### 2.2. *In Vitro* Angiogenesis Assay

We used an *in vitro* angiogenesis assay (endothelial tubule formation in Matrigel) to test the capacity of ECFC to form capillary tubule-like networks. In a 96-well plate, 17,000 cells per well were seeded in 150 *μ*l treatment medium with 30 *μ*l growth factor reduced in Matrigel (BD Biosciences, Bedford, MA). ECFCs were either treated with 0.5% and 1% ethanol for the duration of the assay or pretreated for 24 h with 0.5% and 1% ethanol. A corresponding control w/o ethanol was run in tandem. After 16 h of incubation at 37°C and 5% CO_2_ supply, each well was photographed with a LEICA DMI 6000 B microscope. Total tubule length in each visual field was measured using the ImageJ software 1.52q (National Institutes of Health). All experiments were performed in triplicate wells from which values were averaged (*n* = number of experiments).

### 2.3. Cell Migration Assay

To analyze ECFC migratory ability, 50,000 ECFCs were seeded in each well of a 12-well plate with a growth medium containing 2.44% supplements, 5% FCS, and 1.2% penicillin/streptomycin. After reaching confluence, the ECFC monolayer was scratched using a sterile P200 pipette tip to produce a lane free of cells as described before [20]. ECFCs treated with 0.5% and 1% ethanol or after 24 h of preincubation with 0.5% or 1% ethanol and a corresponding control w/o ethanol were run in tandem. Light microscopic images were obtained immediately after the scratch (T0) and at the end of the experiment after 18 h (T18). Migration into the scratch wound was analyzed using the ImageJ software and calculated as the percentage of wound closure (percentage of original area at T0 that became occupied by cells by migration into the wound area at T18). All experiments were done in quadruplicate wells from which values were averaged.

### 2.4. Cell Impedance Assay

Cell impedance was determined by real-time impedance analysis using the xCelligence Real-time analyzer (RTCA, Roche, Mannheim, Germany). The Cell Index (CI), which reflects cell adherence, is converted from impedance measurement by the xCelligence software (Version 1.2.1) and was continuously monitored every 20 min for at least 72 h and directly after specific treatments of the cells. For experiments done, 10,000 cells were seeded in triplicates onto gold-coated E-Plate VIEW 96-well plates (Roche, Mannheim, Germany) and proliferation was calculated through measuring increasing impedance. ECFCs treated with 0.5% and 1% ethanol or after 24 h of preincubation with 0.5% or 1% ethanol and a corresponding control w/o ethanol were run in tandem.

### 2.5. Cell Proliferation Assay

To determine the proliferative capacity of ECFCs after treatment with ethanol, 50,000 cells were seeded per well of 6-well culture plates in EGM supplemented with 5% (*v*/*v*) FBS and 1% penicillin/streptomycin. After 24 h, 48 h, and 72 h of treatment, the cell number was counted in a Neubauer chamber with 1 : 2 trypan-blue dilution. Population doubling time was calculated as the following: log2/(log (Nt/No)/*t*), *t* = time period (h), Nt = number of cells at time *t*, and No = initial cell number. ECFCs after treatment with 0.5% EtOH 24 h and 1.0% EtOH and a corresponding untreated control were run in tandem.

### 2.6. Flow Cytometry

Flow cytometry analysis was performed to detect adhesion molecule expression on the ECFC surface. ECFCs treated with 1% ethanol and a corresponding control w/o ethanol were run in tandem. Cells were harvested by incubating with Accutase (Capricorn, Ebsdorfergrund, Germany) for 10-15 min at room temperature. After washing with flow cytometry buffer (PBS, 2% BSA (Merck, Darmstadt, Germany)), 1 × 10^5^ cells were blocked with normal IgG (Grifols, Paris, France) (5 mg/ml) for 1 min, followed by incubation with the appropriate antibodies (VCAM1 APC (BioLegend, San Diego, CA), PECAM1 FITC (BioLegend, San Diego, CA), and VE-cadherin PE (BioLegend, San Diego, CA)) and isotype controls at 4°C for 30 minutes. For each experiment, a positive control for apoptotic cells was included to exclude dead cells from analysis. Apoptosis was induced by UV-irradiation with a transilluminator (Biostep, Jahnsdorf, Germany) for 30 min, and cells were further incubated for 2 h at room temperature. Cells were harvested, washed, and resuspended in Annexin V binding buffer, stained with Annexin V FITC (BioLegend, San Diego, CA) and incubated for 20 min. After washing and prior to measurement, propidium iodide (10 *μ*g/ml) (Sigma-Aldrich, Darmstadt, Germany) was added. Flow cytometry measurements were performed on a BD FACSCalibur Flow cytometer (Becton Dickinson, Heidelberg, Germany), and results were analyzed using the FlowJo X Software V10 (Tree Star, Ashland, OR).

### 2.7. RT-PCR for Quantification of VEGF, sFLT-1, PlGF, and sEng Gene Expression

The RNA isolation of ECFC was performed according to the protocol of Chomczynski et al. which was slightly modified [21]. ECFCs treated with 1% ethanol and a corresponding control w/o ethanol were run in tandem. The concentration of RNA of each sample was determined spectrophotometrically (BIO photometer, Eppendorf, Germany). For the cDNA synthesis, 2 *μ*g of RNA was diluted with diethylpyrocarbonate (DEPC) water to a volume of 8 *μ*l and denatured at 68°C for 10 min in a thermocycler (PTC 200, Biozym Scientific GmbH, Germany). Then, 12 *μ*l of High Capacity cDNA Reverse transcription (RT) master mix was added. In each case, 1.5 *μ*l diluted cDNA and 10.5 *μ*l master mix were pipetted into the appropriate strip tubes (0.1 ml). For each treatment, triplets were created and three RT-PCR runs were done for each patient. For normalization, 18S rRNA served as a housekeeping gene. Primer sequences used were as follows: VEGF-A forward primer 5-TACCTCCACCATGCCAAGTG-3, VEGF-A reverse primer 5-GATGATTCTGCCCTCCTCCTT-3, sFlt1 forward primer 5-AATCAGAGGTGAGCACTGCAAC-3, sFlt1 reverse primer 5-TGGTACAATCATTCCTTGTGCTTT-3, PlGF forward primer 5-CCTACGTGGAGCTGACGTTCT-3, PlGF reverse primer 5-CCTTTCCGGCTTCATCTTCTC-3, sEng forward primer 5-ACCTTTGGTGCCTTCCTCAT-3, sEng reverse primer 5-CAATCCCTCAGAGGCTTCAC-3, 18S rRNA forward primer 5-ACATCCAAGGAAGGCAGCAG-3, and 18S rRNA reverse primer 5-TTTTCGTCACTACCTCCCCG-3. Relative expression levels of ECFC from control vehicle and incubation with ethanol were finally compared.

### 2.8. Immunoblot for Quantification of VEGF, sFlt-1, and VE-Cadherin

For analysis of proteins VEGF, sFlt-1, and VE-cadherin, ECFCs were grown to 50% confluence in 100 mm dishes (Sarstedt, Nümbrecht, Germany) in an endothelial growth medium with 2.5% FCS with the respective additives. ECFCs were seeded into 100 mm dishes and treated with either only EGM (C = control) or EGM plus 1% ethanol. Cells were treated with 6 ml medium and cultivated for 24 h at 37°C, followed by harvesting of the cells, cell lysis, and protein quantification.

Protein lysates were separated by SDS-PAGE and transferred to nitrocellulose membranes (GE Healthcare, Braunschweig, Germany). After blocking for 1 h with 5% dry milk powder in PBS-T, the membrane was incubated overnight at 4°C with PBS-T plus 5% dry milk powder and appropriate antibodies: 1 : 1000 anti-VEGF (Abcam, Cambridge, UK), 1 : 400 anti-sFlt-1 (Sigma-Aldrich, St. Louis, Missouri, USA), and 1 : 2000 anti-VE-cadherin (Abcam, Cambridge, UK). After three times of washing with PBS-T, the secondary antibody was added (1 : 5000 goat anti-rabbit; GE Healthcare, Braunschweig, Germany) in 5% dry milk powder in PBS-T for 2 h at room temperature. Visualization of immunoblot bands was performed by using ECL chemiluminescence (Pierce) and X-ray. The X-rays were scanned and analyzed with the ImageJ software.

### 2.9. ELISA for Quantification of Secreted Protein Levels of PlGF and sEng

PlGF and sEng concentrations were measured by enzyme-linked immunosorbent assay (ELISA) (PlGF & sEng Quantikine ELISA Kit R&D Systems, United States) according to the manufacturer's recommendations. Briefly, 100 *μ*l of concentrated cell culture supernatant for PlGF or 50 *μ*l for sEng was added per well. Standards and controls were assayed in duplicates and samples in triplicates and after incubation with the appropriate conjugates measured by using a microplate reader (Thermo Fisher Scientific) at 450 nm.

### 2.10. Statistical Analysis

The collected individual measured values (*n*) from experiments were analyzed with GraphPad Prism7 (GraphPad Software Inc.) for their statistical relevance with a Mann-Whitney *U*-test or unpaired *t*-test after determination of data distribution using the Shapiro Wilk normality test. Data are shown as mean and standard deviation (SD). A significant deviation of the value pairs is indicated by a *P* value (<0.05) and by one asterisk (∗).

## 3. Results

### 3.1. *In Vitro* Angiogenesis

**Figure 1:**
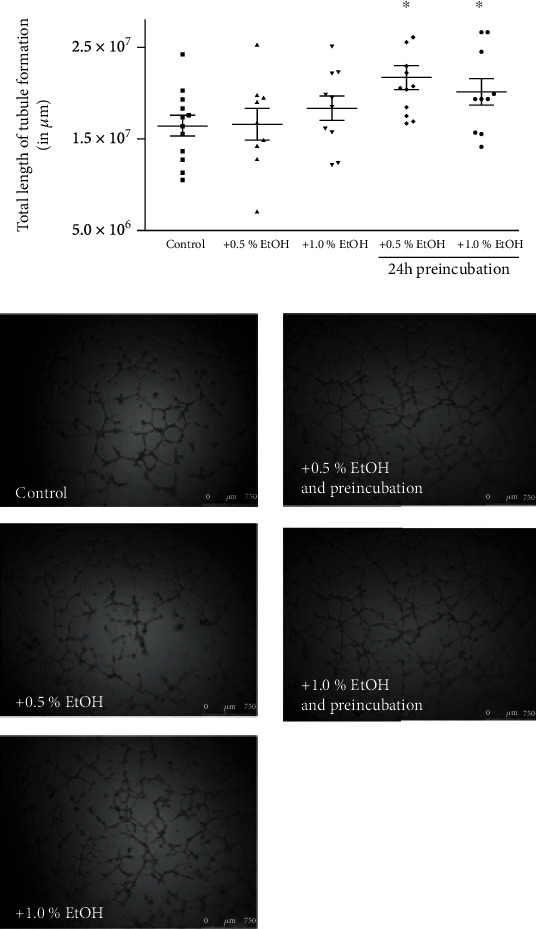
Effect of ethanol on capillary-tube formation in a Matrigel assay. ECFCs were cultured in endothelial basal medium ((EBM) +5% FBS) and treated with 0.5% or 1% ethanol directly prior assay start or 24 h before or without ethanol (control). Capillary-tube formation (average total tubule length per microscopic field) was analyzed after 16 h by visual microscopy at 2.5x magnification. Data are expressed as total tubule length in *μ*m. Results represent mean values of total tubule length ± SD of at least 6 independent experiments. ^∗^*P* < 0.05 vs. untreated control.

A Matrigel angiogenesis model was used to assess the capacity of ECFC to differentiate into tubule-like structures (Figure 1). Tubule assemblage by ECFC pretreated for 24 h with ethanol was markedly higher in comparison to untreated ECFC (control: 1.64∗10^7^ ± 4.0∗10^6^ *μ*m; 0.5% ethanol: 2.17∗10^7^ ± 4.4∗10^6^ *μ*m, *P* = 0.001; 1.0% ethanol: 2.01∗10^7^ ± 4.5∗10^6^ *μ*m, *P* = 0.03). In the absence of preincubation, ethanol did not impact tubule formation (0.5% ethanol: 1.66∗10^7^ ± 5.2∗10^6^ *μ*m, *P* = 0.36; 1.0% ethanol: 1.84∗10^7^ ± 4.3∗10^6^ *μ*m, *P* = 0.48).

### 3.2. Migration

**Figure 2:**
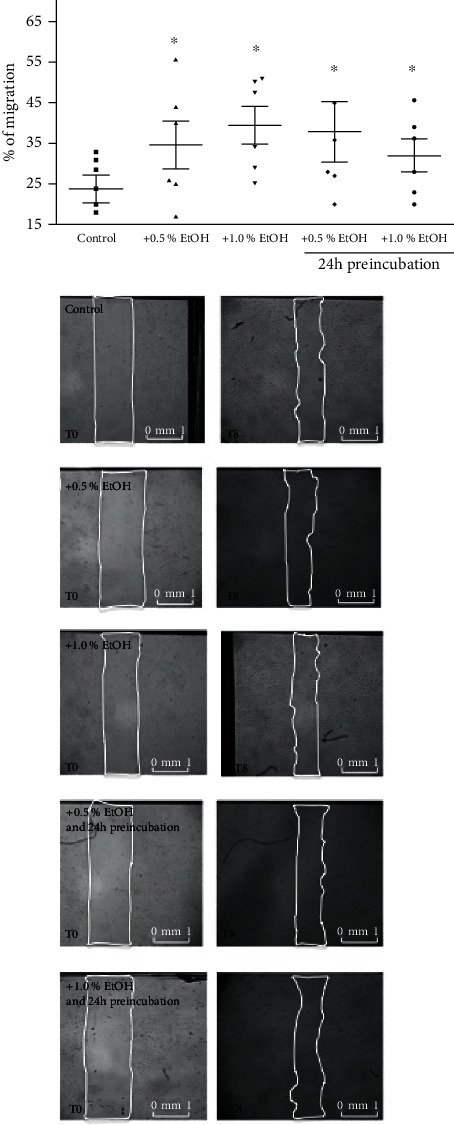
Effect of ethanol on ECFC migration. ECFCs were cultured in endothelial basal medium (EBM) +5% FBS and in the absence or presence of 0.5% or 1% ethanol directly prior assay start or 24 h before. The migration of ECFC into the scratch wound was assessed after incubation for 8 h. Results of at least 6 independent experiments represent mean ± SD percent wound filling. ^∗^*P* < 0.05 vs. untreated control.

With scratch wound area filling expressed as percent of total possible lane closure (100%), the migration of ECFC in the presence and absence of ethanol was tested (Figure 2). Migration by ethanol-treated ECFC was markedly higher in comparison to untreated control (control: 22.6% ± 8.7%; 0.5% ethanol: 31.4% ± 11.5%, *P* = 0.03; 1.0% ethanol: 37.4% ± 11.5%, *P* = 0.001; 24 h preincubation 0.5% ethanol: 34.6% ± 13.8%, *P* = 0.01; 24 h preincubation 1.0% ethanol: 29.8% ± 8.8%, *P* = 0.004).

### 3.3. Cell Impedance

**Figure 3:**
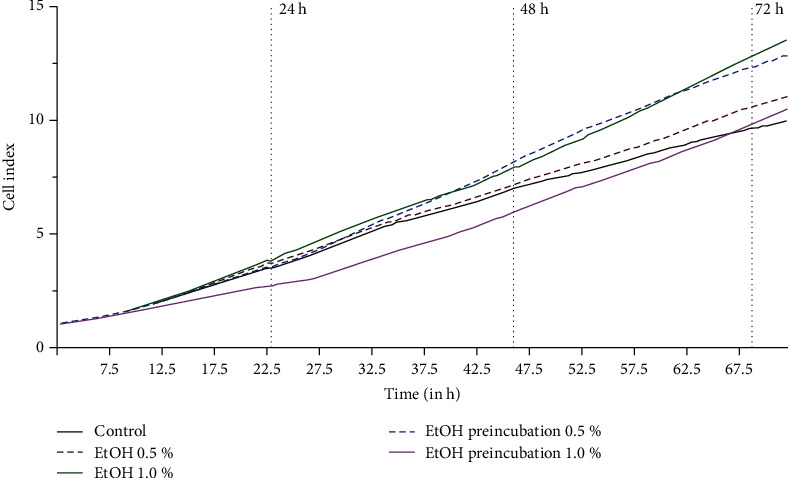
Effect of ethanol on ECFC cell impedance. ECFCs were incubated in the absence or presence of ethanol (0.5% or 1% or 24 h of preincubation with 0.5% or 1% ethanol) in EGM +5% (*v*/*v*) FBS. Cell impedance was measured after 72 h for at least 6 independent experiments.

To compare the cell impedance of ECFC treated with ethanol and controls, changes in impedance were determined. Cell impedance of ethanol-treated ECFC was measured up to 72 h and was markedly higher in comparison to untreated control (*P* values at timepoint 72 h vs. control: 0.5% ethanol: *P* = 0.004, 1% ethanol: *P* = 0.001; preincubation 0.5% ethanol: *P* = 0.01; preincubation 1% ethanol: *P* = 0.64) (Figure 3).

### 3.4. Proliferation

**Figure 4:**
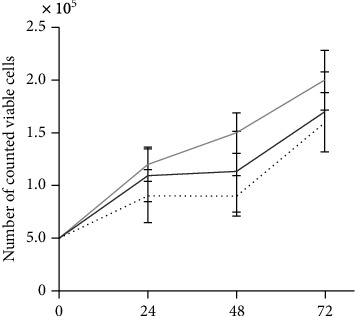
Effect of ethanol on ECFC proliferation rate. ECFCs were incubated in the absence or presence of ethanol (0.5% or 1% ethanol) in EGM +5% (*v*/*v*) FBS. Cell numbers were counted, and population doubling time was calculated after 24 h for at least 4 independent experiments.

The population doubling time of ECFC was shorter in ethanol-treated cells even though not significantly different (control: 24.91 h; 0.5% ethanol: 20.75 h; *P* = 0.43 vs. control, 1% ethanol: 17.42 h; *P* = 0.44 vs. control) (Figure 4).

### 3.5. VE-Cadherin, VCAM, and PCAM Expression

**Figure 5:**
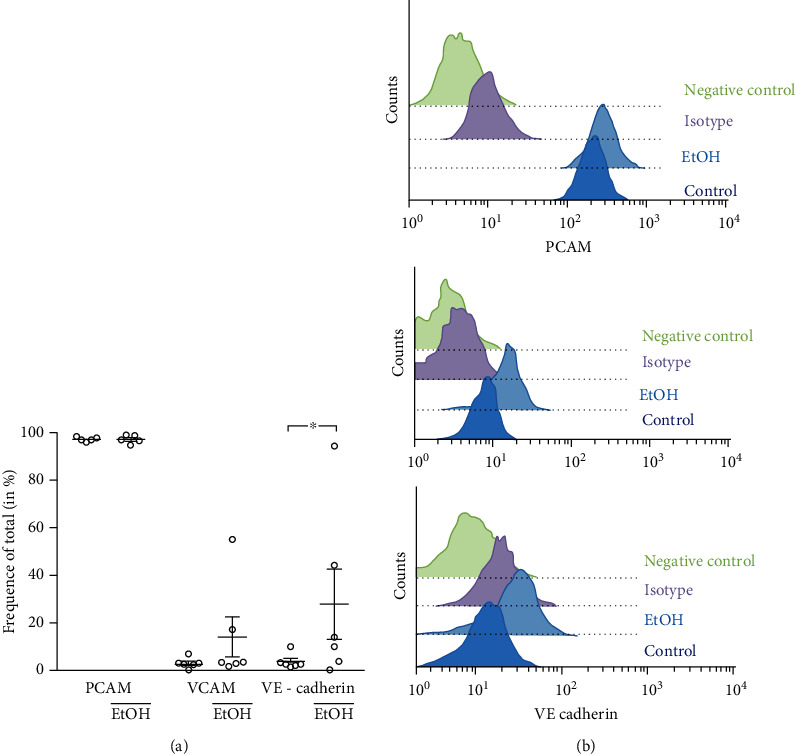
Ethanol-dependent ECFC cell surface expression of VE-cadherin, VCAM, and PECAM1. (a) ECFCs were treated with or without 1% ethanol and incubated with antibodies (anti-VE-cadherin, anti-VCAM, and anti-PECAM1) and analyzed by flow cytometry. Expression levels of VCAM, VE-cadherin, and PCAM are shown. Results of at least 8 independent experiments represent cell surface expression in % shown as mean ± SD. ^∗^*P* < 0.05 vs. untreated control. (b) Flow cytometry analysis of PCAM, VCAM, and VE-cadherin on ECFC cell surface after treatment with EtOH.

Using flow cytometry, we tested the ethanol-dependent cell surface expression of VE-cadherin, VCAM, and PCAM on ECFC (Figure 5). The expression of VE-cadherin by ethanol-treated ECFC was significantly higher in comparison to untreated control (control: 3.77% ± 3.1%; 1% ethanol: 27.9% ± 15.0%, *P* = 0.01) whereas VCAM and PCAM expression was not different (VCAM: control: 3.2% ± 2.2%; 1% ethanol: 14.0% ± 10.1%, *P* = 0.31; PCAM: control: 97.3% ± 1.0%; +1% ethanol: 97.2% ± 1.8%, *P* = 0.96).

### 3.6. VEGF, sFLT-1, PlGF, and sEng Gene Expression

**Figure 6:**
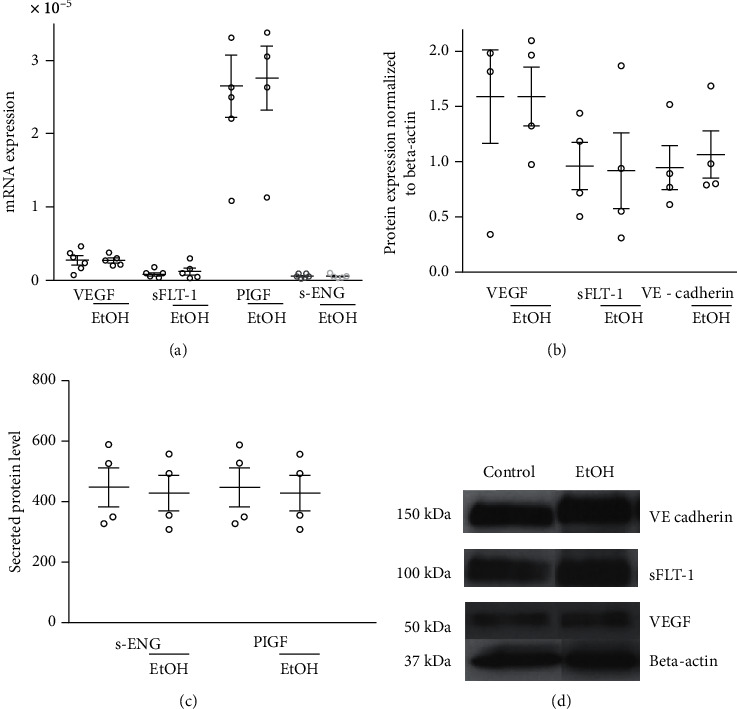
Validation of ethanol-dependent expression of VEFG, PlGF, sFlt-1, sEng, and VE-cadherin. Gene expression levels, protein expression, and secreted protein levels of ECFC from untreated ECFC or after treatment with 1% ethanol were determined. For each experimental group, triplicates were created and RT-PCR runs (a), immunoblot (b, d), and ELISA (c) were performed for each ECFC cell line. Results represent mean values ± SD of at least 6 independent experiments.

There was no significant difference in the VEGF, sFLT-1, PlGF, or sEng gene expression of ECFC treated with ethanol in comparison to vehicle control (Figure 6(a): VEGF: *P* = 0.60; sFlt-1: *P* = 0.26; PlGF: *P* = 0.93; sEng: *P* = 0.96).

### 3.7. Protein Expression Level of VEGF, sFlt-1, VE-Cadherin, and Secreted Protein Levels of sEng and PlGF

There was no significant difference in the protein expression level of VEGF, sFlT-1, or VE-cadherin of ECFC treated with ethanol in comparison to vehicle control (Figure 6(b): VEGF: *P* = 0.99; sFlT-1: *P* = 0.86; VE-cadherin: *P* = 0.75). Also, the secreted protein level of sEng and PlGF did not differ between ethanol-treated cells and vehicle control (Figure 6(c): sEng: *P* = 0.13; PlGF: *P* = 0.13).

## 4. Discussion

In this study, we demonstrate a promoting effect of moderate ethanol concentrations on angiogenic capacities of ECFC. In this context, we tested the ability of migration, proliferation, and tubule formation, which were enhanced after incubation with moderate concentrations of ethanol. These functional properties represent cell characteristics important for angiogenesis and vasculogenesis, and they are markers of vascular health. Ethanol-treated cells showed a higher expression level of the cell surface marker VE-cadherin, a protein which plays a significant role in cell-cell interaction. Interestingly, the positive effect of ethanol seems not to be triggered by VEGF, PlGF, sEng, or sFlt-1. Additionally, expression levels of their mRNAs, protein, or soluble protein expression were not different after ethanol treatment compared to untreated control.

Our findings confirm data of previous studies in which a stimulating effect of ethanol on endothelial function is demonstrated [26, 27]. To our knowledge, however, this is the first study to demonstrate enhanced functional properties of ECFC treated with ethanol. ECFCs are an endothelial cell type with a strong intrinsic clonal proliferation potential and the ability to contribute to de novo blood vessel formation *in vitro* and *in vivo* [21, 22]. The ECFC recruited into the damaged tissue was derived either from the circulation or from the local vascular wall [23]. The endothelial integrity of the vascular wall is restored by migration and proliferation of ECFC [24, 25]. Taken together, based on the current accumulated evidence, ECFCs are the most rational and promising cell sources that are able to incorporate into or form vessels directly in areas of tissue regeneration.

There are some epidemiological studies that demonstrate the benefits of moderate ethanol consumption in ischemic heart disease [5–7]. Interestingly, the results of these works suggest that in addition to lowering the rate of atherosclerosis through regular exposure to ethanol over many years, at least part of the ethanol-induced protection of the vascular endothelium can be induced within a few minutes by a short exposure. This protection can be provided by moderate ethanol doses, which correspond to about one or two alcoholic beverages. At this juncture, the concentrations of ethanol used in this study, i.e., 0.5% (17 mM) and 1% (34 mM), are levels that do not cause intoxication *in vivo* and correspond to 2 to 3 standard drinks per day [28]. Chen et al. showed that the treatment of cardiac muscle cells with 10-50 mM ethanol protects against ischemia by activating protein kinase C [26]. The ability of ethanol to activate protein kinase C has also been observed in several other cell systems [29, 30] and has been reported to mediate the protection of the heart from ischemia. *In vivo* studies demonstrated cardiovascular protection after prolonged ethanol administration of 25-50 mM also in a guinea pig model.

Restoring blood flow after tissue injury or occlusion requires angiogenic germination of endothelial cells from nearby intact blood vessels, as well as vasculogenesis through circulating ECFC to allow invasion of new blood vessels to restore tissue perfusion. A short-term treatment with ethanol to increase the capacity of ECFC could be a possible therapeutic approach to enable the blood flow to be restored more quickly.

The exact reasons why ethanol can protect the cardiac tissue and endothelium have not yet been fully clarified. It is certain that ethanol can change the biophysical and biochemical properties of cell membranes. Ethanol interacts directly with membrane proteins to modulate their activity. In different cell models, exposure of ethanol impacted both the signal transduction mediated by protein kinase C [31, 32] and the cAMP-dependent protein kinase [30, 33].

In our study, we show an increased expression of the membrane surface molecule VE-cadherin, which is significant for cell-cell interaction. Processes, which are mediated by adhesion molecules, are activation of cell mobilization, migration, and proliferation via loosening of endothelial cell adhesion complexes. These mechanisms are impaired in CVD [17, 34]. Widner et al. showed that the expression of VE-cadherin is reduced in plaque microvessels leading to vascular damage [35]. The observed increase of VE-cadherin in our study might contribute to an increased repair capacity of the endothelium.

With regard to the cardioprotective effect, half a million adults and 15000 children undergo open-heart surgery annually in the United States each year in which the heart is exposed to controlled periods of ischemia. Despite advances in heart protection, myocardial dysfunction remains a major cause of morbidity and mortality in the immediate postoperative period. The exact time of the expected ischemia is known in this context. Therefore, the targeted use of a cardioprotective agent can prevent the consequences of ischemia in the endothelium and the remaining heart tissue. One could speculate that moderate doses of ethanol could be cardioprotective and this approach could be evaluated further.

ECFCs seem to be a promising cell source for revasculogenesis in damaged tissue in CVDs. They have excellent vascularization potential *in vitro* and *in vivo*, and in this study, we show that their potential is further increased by adding small amounts of ethanol. ECFC can be isolated in sufficient numbers from umbilical cord blood for possible use in newborns but if stored long-term could be used as an autologous source of reparative cells for the treatment of CVDs in adulthood. In older children and adults, primarily circulating peripheral blood ECFCs are less clonogenic, proliferative, and angiogenic than umbilical cord ECFC. Finally, it is known that certain disease states such as diabetes can reduce the frequency and function of the isolated ECFC to such an extent that these cells are not of sufficient quality for use as revascularization therapy [36, 37]. Here, short-term therapy with ethanol could possibly increase the capacity and proliferation power of the ECFC in order to achieve a higher quantity and quality of these cells.

In conclusion, ethanol at moderate concentrations enhances the angiogenic abilities of ECFC. Ethanol-exposed cells showed a higher expression level of VE-cadherin, suggesting an increased endothelial repair capacity. Our findings support data from epidemiological studies demonstrating the benefits of short exposure of ethanol to prevent cardiovascular damage in adults [5–7]. However, the data provided here are limited due to the use of primary cells under *in vitro* conditions and show the short-term effect of ethanol on endothelial functionality. Further studies on the impact of long-term moderate ethanol exposure on ECFC biology are still needed.

## Data Availability

The experimental data and row data used to support the findings of this study are available from the corresponding author upon request.
